# Genetic structure of recently fragmented suburban populations of European stag beetle

**DOI:** 10.1002/ece3.6858

**Published:** 2020-10-02

**Authors:** Karen Cox, Niall McKeown, An Vanden Broeck, An Van Breusegem, Roger Cammaerts, Arno Thomaes

**Affiliations:** ^1^ Research Institute for Nature and Forest (INBO) Geraardsbergen Belgium; ^2^ Institute of Biological, Environmental and Rural Sciences (IBERS) Aberystwyth University Aberystwyth UK; ^3^ Retired from the Natural and Agricultural Environment Studies Department (DEMNA) Public Service of Wallonia Gembloux Belgium; ^4^ Research Institute for Nature and Forest (INBO) Brussels Belgium

**Keywords:** bottleneck, connectivity, land use change, *Lucanus cervus*, sex‐biased dispersal, suburban populations

## Abstract

Habitat loss and fragmentation due to urbanization can negatively affect metapopulation persistence when gene flow among populations is reduced and population sizes decrease. Inference of patterns and processes of population connectivity derived from spatial genetic analysis has proven invaluable for conservation and management. However, a more complete account of population dynamics may be obtained by combining spatial and temporal sampling. We, therefore, performed a genetic study on European stag beetle (*Lucanus cervus* L.) populations in a suburban context using samples collected in three locations and during the period 2002–2016. The sampling area has seen recent landscape changes which resulted in population declines. Through the use of a suite of *F*_ST_, clustering analysis, individual assignment, and relatedness analysis, we assessed fine scale spatiotemporal genetic variation within and among habitat patches using 283 individuals successfully genotyped at 17 microsatellites. Our findings suggested the three locations to hold demographically independent populations, at least over time scales of relevance to conservation, though with higher levels of gene flow in the past. Contrary to expectation from tagging studies, dispersal appeared to be mainly female‐biased. Although the life cycle of stag beetle suggests its generations to be discrete, no clear temporal structure was identified, which could be attributed to the varying duration of larval development. Since population bottlenecks were detected and estimates of effective number of breeders were low, conservation actions are eminent which should include the establishment of suitable dead wood for oviposition on both local and regional scales to increase (re)colonization success and connectivity among current populations.

## Graphical abstract

In this genetic study of suburban European stag beetle populations, we investigated the influence of recent habitat fragmentation using spatial and temporal samples. The results suggest the populations to be currently demographically independent, with dispersal being mainly female‐biased. Since recent bottlenecks were detected and estimates of effective number of breeders were low, conservation actions are eminent.

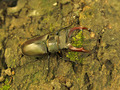

## 1 INTRODUCTION

Land use changes such as urbanization have significantly driven habitat loss and population fragmentation. Such fragmentation can reduce gene flow among populations and decrease population sizes. This can in turn increase the rate at which genetic variation is lost by genetic drift and the negative effects of inbreeding depression on survival and reproduction, ultimately increasing the risk of population extinction (Allendorf et al., 2013). In contrast, population viability may be enhanced by connectivity to surrounding populations. Inference of patterns and processes of population connectivity derived from spatial genetic analysis has proven invaluable for conservation and management (van Strien et al., 2014). However, a more complete account of population dynamics may be obtained by combining spatial and, often neglected, temporal sampling. Firstly, where genetic differences are low, temporal stability of the differentiation adds confidence that this reflects a true signal (Waples, 1998). Second, the use of temporal samples is one of the best ways to estimate the crucial demographic factor effective population size. Finally, analysis of temporal samples permits recognition of population turnover (i.e., when local populations become extinct, but the habitat patch is recolonized). Discriminating between situations whereby genetic differences reflect permanent population isolation or metapopulation dynamics (i.e., dynamic balance between migration, extinction, and recolonization) is important for judicious conservation, particularly for species with low colonizing ability (Holyoak & Ray, 1999).

**FIGURE 1:**
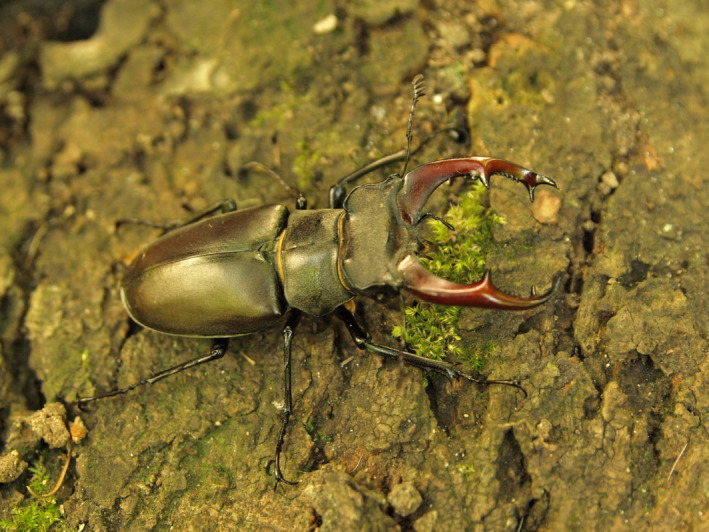
Male European stag beetle at Jagersveld, Watermaal‐Bosvoorde (Belgium)

The European stag beetle, *Lucanus cervus* L. (Coleoptera: Lucanidae; Figure 1), is a saproxylic beetle attributed with limited vagility. Although it is distributed widely across Europe, a decline is presumed in many countries and regions, and populations have gone extinct or are threatened at the northern edge of its range (Harvey et al., 2011). Accordingly, the species has been designated “near threatened” in the European Red List (Cálix et al., 2018) and is protected by the Habitats Directive of the European Union since 1992 and by the Bern Convention since 1979 (Luce, 1996). Habitat loss and fragmentation have been identified as the main threats for this species (Della Rocca et al., 2017). Moreover, habitat continuity was found to be of major importance to maintain its populations (Thomaes, 2009; Thomaes et al., 2018).

Stag beetle is a semelparous species with multiple oviposition events in the same season (Tini et al., 2018). Larval development goes through three instar stages (Fremlin & Hendriks, 2014) and takes approximately three to six years, depending on the area within its distribution range (Fremlin & Hendriks, 2014; Harvey et al., 2011; Hawes, 2009; Hendriks & Méndez, 2018; Rink & Sinsch, 2008). Emergence can be observed from May to June, and adults are active for a few weeks up to three months (Harvey et al., 2011). Although the life cycle of stag beetle suggests its generations to be discrete, larval development, especially the third instar stage, can vary in duration, even locally due to the microclimate and habitat quality.

So far, evidence showed that dispersal distances in the species seem to range from a few hundred meters up to a maximum of five kilometers (Rink & Sinsch, 2007). As female stag beetles usually stay close to their site of emergence, males are considered the main dispersers (Rink & Sinsch, 2007; Tini et al., 2017, 2018). Female dispersal distances become even smaller when suitable oviposition sites are abundant and nearby, according to Tini et al. (2018). However, in suburban areas and under less suitable habitat conditions, dispersal distances can increase (Rink & Sinsch, 2007; Thomaes et al., 2018). Sex‐biased dispersal may be scale dependent, since long‐distance and short‐distance dispersal are likely to be caused by different reasons. While inbreeding is likely avoided by dispersal on a local scale (e.g., Lebigre et al., 2010), dispersal over a longer distance could be induced by the need to colonize new sites or by high local competition or predation risk (Lawson Handley & Perrin, 2007).

Dispersal behavior of stag beetle has to date been studied primarily using radio‐telemetry and mark‐and‐recapture methods (e.g., Chiari et al., 2014; Hawes, 2008; Rink & Sinsch, 2007; Thomaes et al., 2018; Tini et al., 2018). These methods, however, are limited due to low sample sizes, short time windows, and difficulties to register long dispersal events. Furthermore, they do not monitor effective dispersal (i.e., dispersal followed by reproduction). Genetic markers have proven useful for the monitoring of “real‐time” dispersal and gene flow over space and time in other systems but have not been applied to many saproxylic species, especially within a fine scale habitat fragmentation context.

The objective of this study was to assess population genetic structure and sex‐specific dispersal among stag beetle within a region that has undergone recent habitat fragmentation linked to urbanization. Until the 60s, stag beetle was quite common in the surroundings of Brussels, capital of Belgium (Thomaes et al., 2008, 2010). As in other regions of NW Europe (Rink & Sinsch, 2006), the relict populations now occur in more suburban habitats such as gardens, parks, tree‐lined lanes, afforested slopes, and edges of large woodlands, such as Sonian Forest (Thomaes et al., 2010). Due to the species’ limited dispersal capacity, we expected to find evidence of restricted gene flow. Analysis of temporal samples was used to investigate if spatial structure was temporally stable, indicating demographically independent populations. Alternatively, genetic change over time could point to demographic instability which may include population turnover events. We tested for the occurrence of recent genetic bottlenecks and reduced effective population sizes that could be considered coincident with census population declines. Based on the results, implications for conservation were discussed.

## 2 MATERIAL AND METHODS

### 2.1 Study area and sampling

**FIGURE 2:**
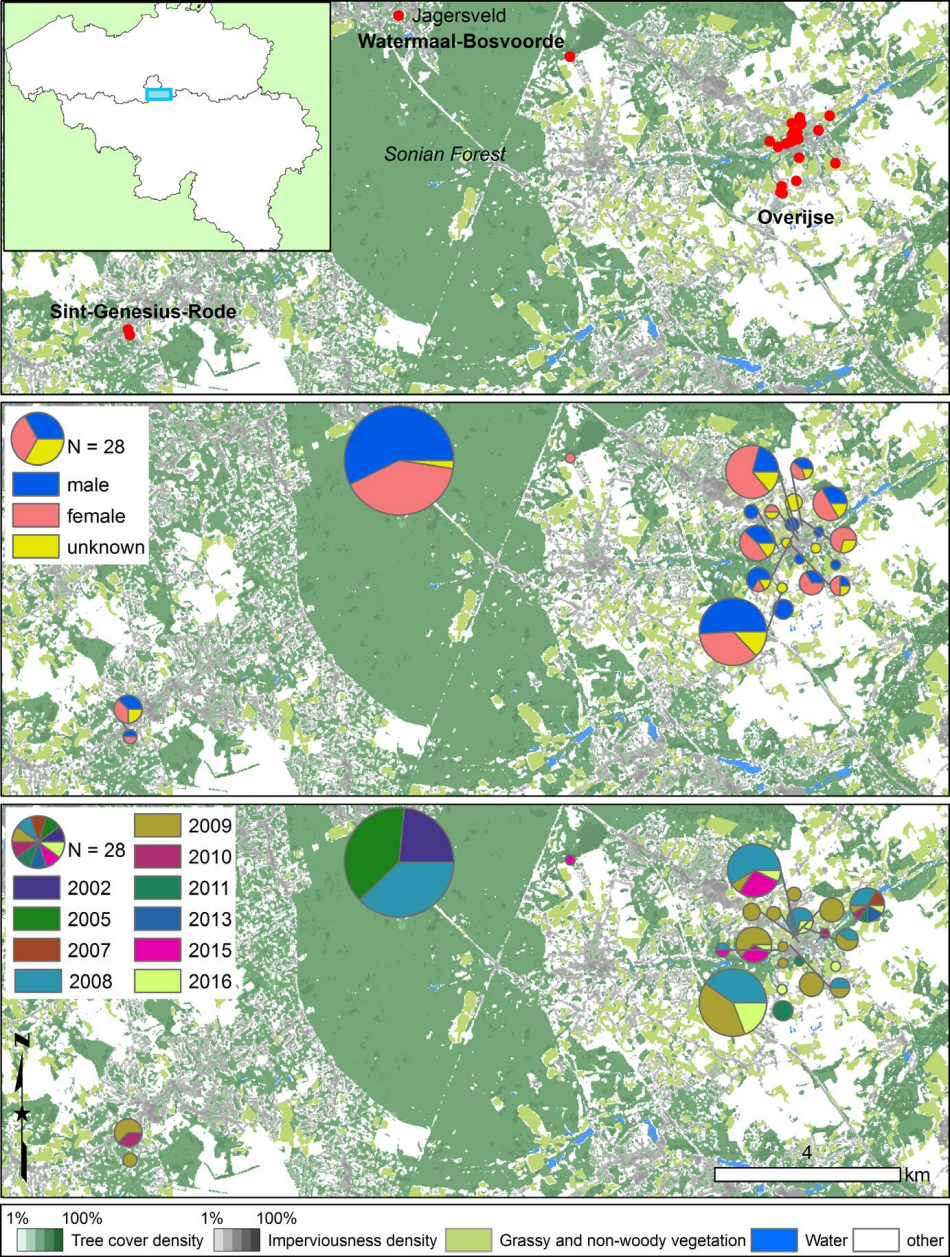
Sampling locations of European stag beetle. Top: The inset shows a regional map of Belgium with the study area indicated with a blue rectangle; the red dots are the sampling locations. Middle: number of females, males, and samples of unknown sex depicted as pie charts. Bottom: number of samples per sampling year depicted as pie charts. The size of the pie charts is relative to the total. Resolution Layers of 2015 with a 20m resolution (https://land.copernicus.eu/pan‐european/high‐resolution‐layers)

The suburban municipalities Beersel, Watermaal‐Bosvoorde, Overijse, and Sint‐Genesius‐Rode hold the remaining populations of *Lucanus cervus* south of the city of Brussels (Belgium) of a once more continuous population from Halle to Leuven (35 km) (Thomaes et al., 2010). This area has been well investigated and most if not all remaining populations are well known (Thomaes, 2009; Thomaes et al., 2010, R. Cammaerts, unpublished data). Jagersveld situated in Watermaal‐Bosvoorde was visited twice per day (by RC), and other sites were visited at least once per week (by AT and a network of volunteers) between the end of May and mid‐September. During each visit, all road casualties and predatory remains ranging from single legs or elytra up to complete beetles were collected starting from 2001 up to 2017 in Jagersveld and from 2007 up to 2017 in the other locations. Sampling permits were granted by the authorities of the Flemish and Brussels Capital Region. A subset of samples from certain years was chosen for genetic analysis depending on the number of samples available per location and per year and on the number of years between sampling, while maximizing the number of different locations where specimens were found (Figure 2, Table 1). For Watermaal‐Bosvoorde, we chose samples taken three years apart starting from 2002 until 2008 (Table 1), which largely concurs with the generation time of stag beetle in the area (Rink & Sinsch, 2008; Smit & Hendriks, 2005). For Overijse, we selected samples taken one to two years apart (Table 1). This variation in number of years between sampling enabled us to test if there is also a temporal genetic structure. Only 10 samples could be collected in Sint‐Genesius‐Rode. A single sample was also collected at the edge of the Sonian Forest near Hoeilaart in 2015. Two small populations in Beersel were not included in this study, located 350 m north and 2 km west of the Sint‐Genesius‐Rode population. Also, a few sites that lie in close vicinity of Jagersveld (Watermaal‐Bosvoorde) have not been sampled. When determinable, the gender of each sampled stag beetle was recorded. The samples were either stored in a freezer (−20°C) or preserved in absolute ethanol and stored in a refrigerator at c. 4°C. Some of the samples were also analyzed for the phylogeographic study of Cox et al. (2019).

**TABLE 1:** List of sampling locations with year of sampling and associated population genetic statistics. When samples were discarded, the adjusted number of samples was given between parentheses

Municipality	Year	*N*	*AR* (*SE*)	*NP*	*H_O_* (*SE*)	*H_E_* (*SE*)	*F*_IS_ (*SE*)
Watermaal‐Bosvoorde (Jagersveld)	2002	32 (29)	2.673 (0.198)	1	0.453 (0.065)	0.491 (0.034)	0.104 (0.086)
Watermaal‐Bosvoorde (Jagersveld)	2005	48	2.686 (0.238)	1	0.451 (0.063)	0.486 (0.041)	0.076 (0.079)
Watermaal‐Bosvoorde (Jagersveld)	2008	47	2.573 (0.218)	0	0.470 (0.060)	0.488 (0.037)	0.055 (0.070)
Watermaal‐Bosvoorde (near Hoeilaart)	2015	1		0			
**Watermaal‐Bosvoorde**		**128 (125)**	**2.804 (0.247)**	**4**	**0.460 (0.061)**	**0.493 (0.036)**	**0.083 (0.074)**
Overijse	2007	2		0			
Overijse	2008	50	3.033 (0.220)	1	0.478 (0.061)	0.524 (0.031)	0.116 (0.072)
Overijse	2009	56	2.950 (0.232)	2	0.487 (0.067)	0.518 (0.034)	0.086 (0.091)
Overijse	2010	2		0			
Overijse	2011	6		0			
Overijse	2013	2		0			
Overijse	2015	13	3.146 (0.233)	3	0.466 (0.073)	0.523 (0.030)	0.172 (0.099)
Overijse	2016	18 (17)	3.035 (0.273)	0	0.445 (0.067)	0.524 (0.038)	0.170 (0.089)
**Overijse**		**149 (148)**	**3.243 (0.261)**	**18**	**0.474 (0.064)**	**0.520 (0.027)**	**0.119 (0.079)**
Sint‐Genesius‐Rode	2009	7	2.750 (0.310)	0	0.458 (0.075)	0.444 (0.054)	−0.029 (0.082)
Sint‐Genesius‐Rode	2010	3		0			
**Sint‐Genesius‐Rode**		**10**	**2.938 (0.295)**	**0**	**0.441 (0.076)**	**0.429 (0.052)**	**−0.005 (0.086)**

Abbreviations: *AR*, rarefied allelic richness with *n* = 14 on the level of sampling year and *n* = 20 on the population level for rarefaction; *F*_IS_, inbreeding coefficient; *H_E_*, expected heterozygosity; *H_O_*, observed heterozygosity; *N*, number of samples; *NP*, number of private alleles; *SE*, standard errors.

Bold values indicate estimates when samples of different years were analyzed together.

### 2.2 DNA extraction and genotyping

The tissue used for DNA extraction depended on availability, but was mostly muscle tissue from legs. We extracted DNA from ground samples with the E.Z.N.A. Forensic DNA Kit (Omega BioTek, VWR, Haasrode, Belgium) or the DNeasy Blood & Tissue Kit (Qiagen, Venlo, the Netherlands). The DNA was eluted in 70 µl AE buffer. The integrity of DNA of 10% of the samples was assessed on 1% agarose gels. DNA quantification was performed with Quant‐iT PicoGreen dsDNA Assay Kit (Life Technologies) using a Synergy HT plate reader (BioTek). Samples were genpotyped at 17 microsatellite loci: Lcerv‐1, Lcerv‐3, Lcerv‐4, Lcerv‐6, Lcerv‐7, Lcerv‐8, Lcerv‐9, Lcerv‐16, Lcerv‐17, Lcerv‐20, Lcerv‐21, Lcerv‐25, Lcerv‐28, Lcerv‐29, Lcerv‐30, Lcerv‐31, and Lcerv‐36, described by McKeown et al. (2018). We included the primer sets in four multiplex PCRs and one simplex PCR as described by Cox et al. (2019) and performed the PCR reaction under the same conditions. Genotyping analysis was achieved using an ABI 3,500 Genetic Analyzer (Applied Biosystems) and with the GENEMAPPER v.4.0 software package. To test for reproducibility, 7% of the samples were blindly replicated two to five times within and across well plates. Samples with fewer than twelve scored loci were discarded. To investigate possible deviations from Hardy–Weinberg equilibrium (HWE), we used the test available in the program GENEPOP 4.6 (Rousset, 2008). GENEPOP was also used to assess the presence of null alleles with the maximum likelihood method following the expectation maximization (EM) algorithm of Dempster et al. (1977), and a second software program, ML‐NULLFREQ (Kalinowski & Taper, 2006). GENEPOP was further used to test for linkage disequilibrium (LD) for each pair of loci. We conducted these tests for each population (location), for each population and year of sampling, and after excluding potential migrants (see further). We implemented a correction for multiple testing with the false discovery rate method (FDR) (Benjamini & Hochberg, 1995) with a nominal level of 5%. Before further analysis was conducted, replicate genotypes were detected because some samples may have been collected from the same individual due to the nature of the samples (prey rests). Only one genotype among replicates was kept in the final dataset.

### 2.3 Spatiotemporal genetic structure and dispersal

We calculated the following estimates of genetic diversity using the R package hierfstat 0.04–22 (Goudet, 2005) for each population and for each year separately as well when sample size was at least seven: rarified allelic richness (*AR*), observed (*H_O_*) and expected heterozygosity (*H_E_*), and the inbreeding coefficient (*F*_IS_). Number of private alleles was estimated using R package poppr 2.8.5 (Kamvar et al., 2014, 2015). Calculations were conducted using R statistical language (R Core Team, 2019). Mean relatedness coefficients of Queller and Goodnight (1989) were also calculated for each spatial group or population within and among sampling years using COANCESTRY 1.0.1.9 (Wang, 2011). The significance of the comparison of relatedness within a sampling year with relatedness among a set of sampling years was assessed by drawing dyads ad random 1,000 times while keeping the properties of the original groups (with e.g., dyads between individuals sampled in 2008 in Overijse as group 1 and dyads between individuals of 2008 and of 2009 in Overijse as group 2). Average relatedness and difference in relatedness among groups is then calculated each time, delivering a distribution with which the observed difference is compared. Genetic differences between samples were also quantified using the R package diveRsity 1.9.90 (Keenan et al., 2013) with *F*_ST_ (Weir & Cockerham, 1984) and *D*_est_ (Jost, 2008). Unbiased confidence intervals of 95% were calculated from 1,000 bootstraps. They were calculated among populations but also among sampling years of the same location, when sample size was at least seven.

Population structure was investigated using the Bayesian program BAPS v. 6.0 which allows the inclusion of individual geographic coordinates as a prior (Cheng et al., 2013; Corander et al., 2008). The program was run ten times for each value of *K *= 1–15. An admixture analysis (Corander & Marttinen, 2006) was performed using 100 iterations, a minimum of three individuals per population, 200 reference individuals for each population, and 20 iterations of reference individuals. We analyzed individual‐based genetic structure with a principal component analysis (PCA) summarizing the allele frequency data. This was followed by a spatial PCA (sPCA), a useful method to find small scale spatial structure (Jombart et al., 2008). Like PCA, this approach is independent from Hardy–Weinberg assumptions or linkage disequilibrium. This multivariate method uses allele frequencies and accounts for their genetic variability and spatial autocorrelation, calculated with *Moran's I* (Moran, 1948, 1950) on the basis of a connection network. We used a distance based connection network, the neighborhood by distance graph, with a minimum distance *d1* of 0 and a maximum distance *d2* of 15 km to create a closed network. Moreover, the sPCA analysis was performed solely on the samples of Overijse where multiple sites could be sampled. This permitted a local spatial genetic analysis. In this case, the maximum distance of the network was 1 km. Global and local spatial structures (i.e., positive and negative spatial autocorrelation, respectively) were tested with 999 permutations and with randomly distributed allele frequencies as the null hypothesis. Analyses were performed with the R package adegenet 2.1.1 (Jombart, 2008). The spatial pattern of genetic variation was also investigated using spatial autocorrelation analyses on a local scale. We assessed the genetic similarity between pairs of individuals at different distance classes jointly for individuals sampled in years 2007, 2008 and 2009 in Overijse. Analyses were performed with SPAGEDI 1.5 (Hardy & Vekemans, 2002) using *Moran's I*, among pairs of individuals using five distances classes: [0–150 m], [150–500 m], [500–1,000 m], [1,000–1,500 m], and [1,500–2,000 m]. Significant deviation of spatial autocorrelation from a random distribution of genotypes was tested by 10,000 random permutations of individual locations for each distance class. The isolation‐by‐distance (IBD) pattern was tested by performing a linear mixed model with maximum likelihood population effects (MLPE) (Clarke et al., 2002) that uses a residual covariance structure to account for the nonindependence of pairwise distances. We used the R package nlme 3.1–127 (Pinheiro et al., 2016) to fit Euclidean genetic distances, calculated with adegenet, and the correlation structure with the help of R package corMLPE (Pope, 2020), using Euclidean geographic distances as the predictor variable. A likelihood‐ratio based pseudo‐*R^2^* based on an improvement from the intercept only model to the fitted model was calculated with the R package MuMIn 1.43.17 (Barton, 2020).

To characterize dispersal between sites assignment tests were performed in GENECLASS2 (Piry et al., 2004) to detect first generation migrants. We only included the data from 2008 and 2009 to be able to include all three populations and to stay as much as possible within one generation. Assignment tests were performed under the criteria *L*_home_ */L*_max_, the ratio of the likelihood of drawing that individual's genotype from the population in which it was sampled and the maximum assignment likelihood calculated for the individual considering all populations (Paetkau et al., 2004). This criterion holds when all subpopulations are sampled. Because there are two small subpopulations near Sint‐Genesius‐Rode not included in this study, the *L*_home_ criterion could be better suited. The Bayesian method of Rannala and Mountain (1997) together with the resampling algorithm of Paetkau et al. (2004) was used to simulating 10,000 individuals (α = 0.01). We further used sibship analyzed with COLONY 2.0.6.5 (Jones & Wang, 2010; Wang, 2004; Wang & Santure, 2009) to discover potential dispersal events across years through the identification of full‐sibs among different years and through the identification of potential parents using different time scales (i.e., differing in number of years between the sampling of parents and of offspring or differing age at maturity). We used the maximum likelihood approach under the assumption of random mating and with the following settings: full likelihood method, polygamous males and females (N. McKeown, unpublished data), medium run length, and three runs. Pairs of individuals were perceived as full‐sibs or parent‐offspring with a minimum probability of 0.95. We first did the analysis with all genotypes defined as offspring (to find full‐sibs among different sampling years). Then subsets were made by location and year for the offspring set, with individuals collected in earlier years (from multiple locations if possible) as potential parents. A minimum of two years and a maximum of 6 years difference among sampling years were used as thresholds to approximate one generation between offspring and parents for each analysis. For years with small sample sizes, samples of several years were analyzed as offspring together: samples of Overijse collected in 2010, 2011 and 2013, and samples of Overijse from 2013, 2015, and 2016 (Overijse 2016 was also analyzed as a separate offspring dataset).

### 2.4 Sex‐biased dispersal

To assess if the dispersal patterns are different for males and females, we limited the data to those individuals of known sex (see Table 3 for numbers of males and females per population and year). Again, we analyzed spatial autocorrelation for individuals sampled during 2007, 2008, and 2009 in Overijse with SPAGEDI as mentioned above, but now for males and females separately. Secondly, we used the following statistical descriptors as implemented in R package hierfstat: *F*_IS_, *F*_ST_, mean corrected assignment index (*mAIc*) and variance of corrected assignment index (*vAIc*) (Goudet et al., 2002). Due to mixture of resident and immigrant individuals in the sample resulting in a Wahlund effect, a larger heterozygosity deficit and higher *F*_IS_ values are expected for the more dispersing sex (Goudet et al., 2002). *F*_ST_ values on the other hand should be lower for the dispersing sex. The assignment index (*AI*) refers to the probability that a multilocus genotype or individual occurs within a sampling locality (Favre et al., 1997; Paetkau et al., 1995). The correction of the *AI* is performed by subtracting population means after log‐transformation to remove population effect that may arise from different levels of genetic diversity in each population. This creates a distribution centered at zero (Favre et al., 1997), with positive values of *AIc* for resident individuals and negative values for potential dispersers (Goudet et al., 2002). The dispersing sex will have lower *mAIc* values and higher *vAIc* values than the philopatric sex. Significance for the comparisons of all four statistics was assessed using 1,000 permutations. We performed the tests on all populations and on each population separately, except for Sint‐Genesius‐Rode due to the small sample size, using samples from all available sampling years, which could lead to using potentially temporally different populations. Also, this set of samples does not comply with the assumption of sampling individuals after dispersal events and before reproduction. We therefore repeated the analysis on partitioned data sets comprising subsets of years. Finally, we calculated relatedness among individuals and the difference in mean relatedness of each sex within locations using COANCESTRY as described above. It is expected that the group with the smallest relatedness coefficient is the dispersing sex.

### 2.5 Effective population size and bottlenecks

There are several methods to calculate effective population size (*N_e_*), each with their own assumptions. Because these assumptions are often violated or because they cannot all be tested for, it is generally recommended to use multiple methods to estimate *N_e_*. In this study, we used two single sample estimators and two temporal methods.

The sibship assignment method in COLONY, a single sample method, was used with the same settings as described earlier. In addition, the linkage disequilibrium method LDNE with a bias correction (Waples & Do, 2008) implemented in NeEstimator 2.01 (Do et al., 2014) was used, assuming random mating. A minimum allele frequency of 0.05 was chosen and jackknife‐based corrected 95% confidence intervals were calculated. As temporal methods, we used the method developed by Jorde and Ryman (2007) implemented in NeEstimator, with parametric 95% confidence intervals. Because the generation time is not known exactly for the species in the study area we used a generation time of 3–4 years. A second temporal method, a maximum likelihood approach, using the program MLNE (Wang, 2001; Wang & Whitlock, 2003) was used. This method takes effects of migration into account, which can bias estimates of *N_e_*. We chose different scenarios where one population was designated as focal population and one of the other two populations as source population, merging data of the different years. For Overijse as source population for Watermaal‐Bosvoorde, sampling years were limited to 2007 until 2010 to approximate the same sampling period. The maximum *N_e_* value was set to 10,000.

We tested if the populations exhibited a signal of recent genetic bottlenecks using the heterozygosity excess and mode shift tests implemented in BOTTLENECK 1.2.02 (Piry et al., 1999). Heterozygosity excess compared to equilibrium expectations was examined using Wilcoxon signed rank tests under different assumed mutation models: the infinite alleles model (IAM) and the Two Phase Mutation model (TPM) with 90% single step mutations. The tests were performed for each population and sampling year separately, with a sample size of minimum 13 samples.

## 3 RESULTS

### 3.1 Genotyping

Only one out of 149 samples from Overijse did not provide a genetic profile of good quality and was discarded (Table 1). Three individuals from Jagersveld sampled in 2002 appeared to be sampled twice, as indicated by identical genotypes. These replicates were discarded as well before further analysis (Table 1). Five out of seventeen loci showed deviations from Hardy‐Weinberg in Overijse and Watermaal‐Bosvoorde: Lcerv‐16, Lcerv‐20, Lcerv‐25, Lcerv‐29, and Lcerv‐30. Locus Lcerv‐7 was also not in HWE in Overijse. This locus and Lcerv‐20, however, did not show any deviations after potential immigrants identified in the BAPS analysis were excluded or when tests were performed on populations for each sampling year separately. According to the GENEPOP results six loci seemed to show high levels of null alleles in the three populations. These were Lcerv‐16, Lcerv‐21, Lcerv‐28, Lcerv‐30, Lcer‐31, and Lcerv‐36 (not in Sint‐Genesius‐Rode) with more than 20% null alleles in each population. The results using ML‐NULLFREQ showed Lcerv‐30 (18%–25% null alleles) and Lcerv‐16 (10%–24% null alleles) to be problematic, even when subdividing populations according to sampling years. Locus Lcerv‐25 appeared to be in significant LD with other loci in both Overijse (with loci Lcerv‐17 and Lcerv‐28) and Watermaal‐Bosvoorde (with Lcerv‐36). We discarded locus Lcerv‐25 and recalculated population differentiation estimates, population structure analysis using BAPS with and without loci Lcerv‐16 and Lcerv‐30 and compared results. The mean error rate per locus was 2%.

### 3.2 Spatiotemporal genetic structure and dispersal

**FIGURE 3:**
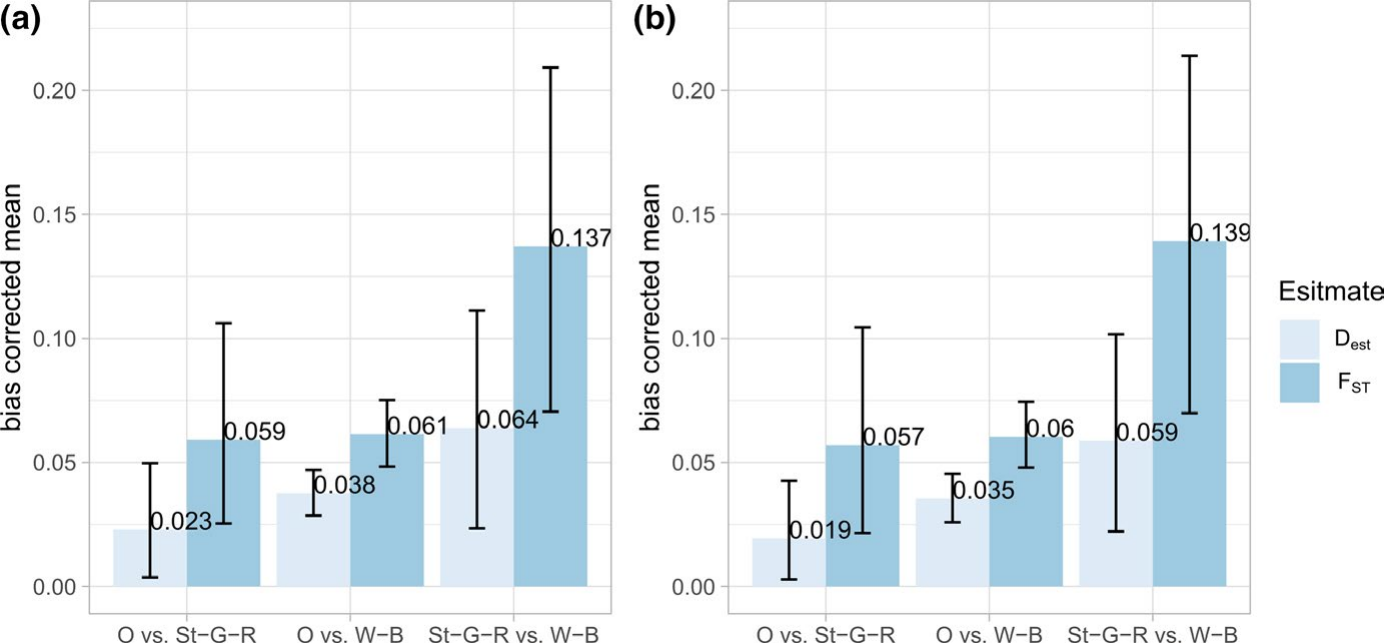
Bias corrected mean pairwise *D*_est_ and *F*_ST_ values with 95% confidence intervals among populations Overijse (O), Sint‐Genesius‐Rode (St‐G‐R), and Watermaal‐Bosvoorde (W‐B) using 17 microsatellite loci (a) and after excluding loci Lcerv‐16 and Lcerv‐30 (b)

Gene diversity was the lowest in Sint‐Genesius‐Rode and the highest in Overijse, although *AR* was quite similar in Sint‐Genesius‐Rode and Watermaal‐Bosvoorde (Table 1). Overijse also exhibited the highest number of private alleles. Despite the overall higher levels of heterozygosity in Overijse, the *F*_IS_ values were slightly elevated in the 2015 and 2016 samples. The other statistics appeared to be stable over the different sampling years. The pairwise values for *F*_ST_ and *D*_est_ are shown in Figure 3a and ranged from 0.059 to 0.137 and from 0.023 to 0.064, respectively. The lowest differentiation values are for the comparisons with Overijse. The pairwise estimates of differentiation changed only very slightly when loci Lcerv‐16 and Lcerv‐30 were excluded (Figure 3b). Differentiation among samples taken in different years in the same location did not differ significantly from zero, except for the *F*_ST_ value among samples from Watermaal‐Bosvoorde in 2002 and samples taken in 2005, which was marginally significant (*F*_ST_ = 0.027; 95% CI = 0.002–0.056). The *D*_est_ value for the same comparison was, however, not significant. Relatedness (*r*) among years was significantly lower than within years for certain cases (Table 2). Most of the significant comparisons were in Watermaal‐Bosvoorde, while almost all comparisons for Overijse were not significant (results not shown), except for the mean difference in *r* in 2009 and among years 2009 and 2015. The latter mean difference in *r* was, however, very small (0.024) and the test was not significant for the 2015 versus 2009–2015 comparison. Likewise in Watermaal‐Bosvoorde, *r* within 2005 was not significantly different from *r* between 2005 and 2008, nor was *r* in 2002 different from *r* between 2002 and 2008, while both among years estimates of *r* differed significantly from *r* of 2008 (Table 2).

**TABLE 2:** Significant mean difference in relatedness within and among years (*p* < .02)

Population	Within year	Among years	*∆r*
Watermaal‐Bosvoorde	2002	2002–2005	0.042
Watermaal‐Bosvoorde	2005	2002–2005	0.043
Watermaal‐Bosvoorde	2008	2005–2008	0.023
Watermaal‐Bosvoorde	2008	2002–2008	0.025
Overijse	2009	2009–2015	0.024

Abbreviation: *∆r*, mean difference in relatedness.

**FIGURE 4:**
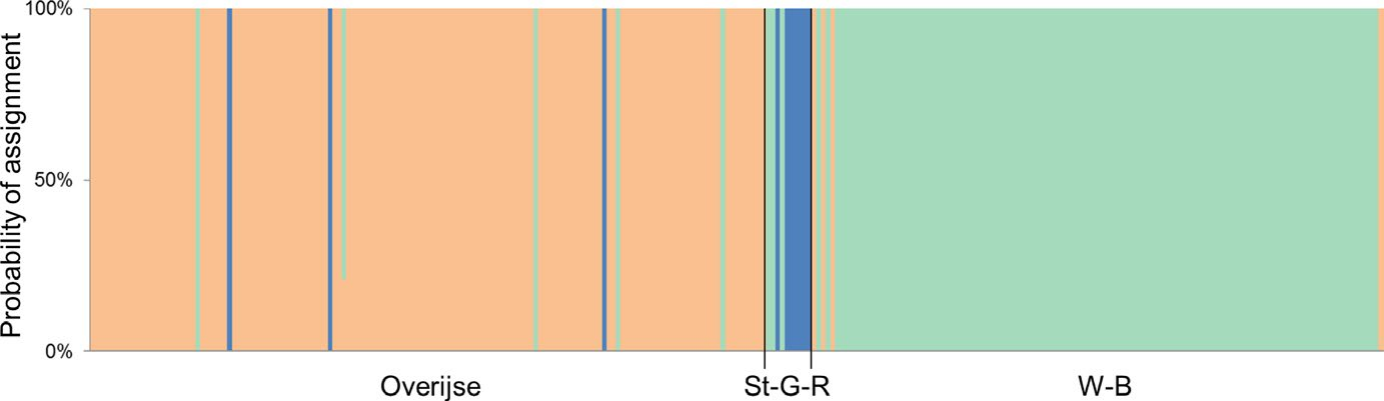
Bar plot of the Bayesian, spatial clustering results using BAPS v. 6.0. Each color indicates a different cluster. The x‐axis gives the three sampling locations: Overijse, Sint‐Genesius‐Rode (St‐G‐R), and Watermaal‐Bosvoorde (W‐B)

The Bayesian, spatial clustering using BAPS and based on 16 microsatellites resulted in three groups, largely dividing the individuals among the sampling locations (Figure 4). Only a few individuals were assigned to another cluster than their sampling location, but there was hardly a sign of admixture, that is, apparently misassigned individuals were migrants rather than hybrids. No clustering according to sampling years was detected. When loci Lcerv‐16 and Lcerv‐30 were excluded, the optimal number of clusters became two as the separation of Sint‐Genesius‐Rode from Overijse resolved. However, differentiation between these sites was evident in *F*_ST_ and sPCA based results.

**FIGURE 5:**
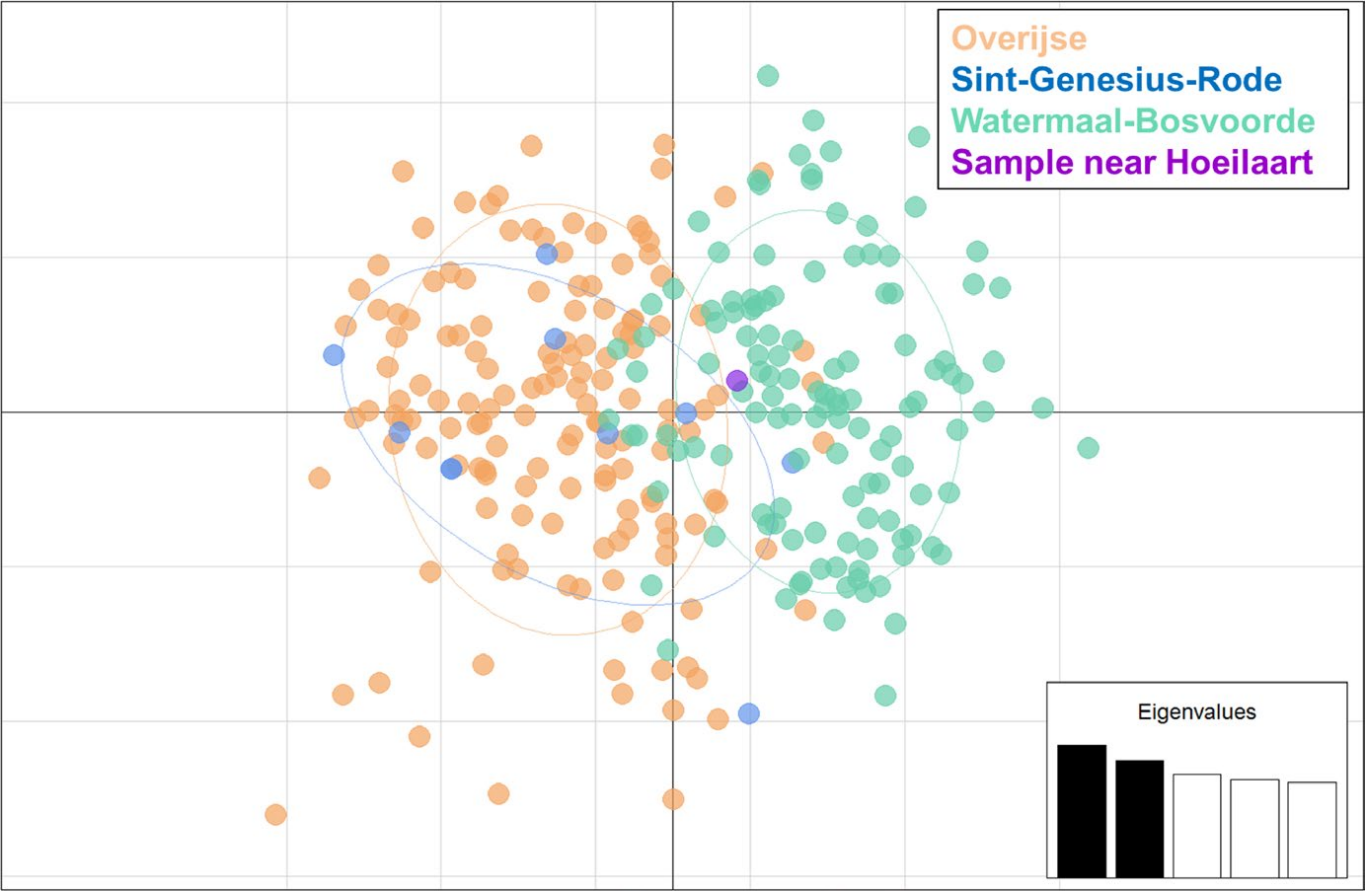
Principal component analysis (PCA) of pairwise genetic distances. The populations are indicated with different colors as given in the legend. The proportion of variance for the first and second principal components is 7% and 5%, respectively

**FIGURE 6:**
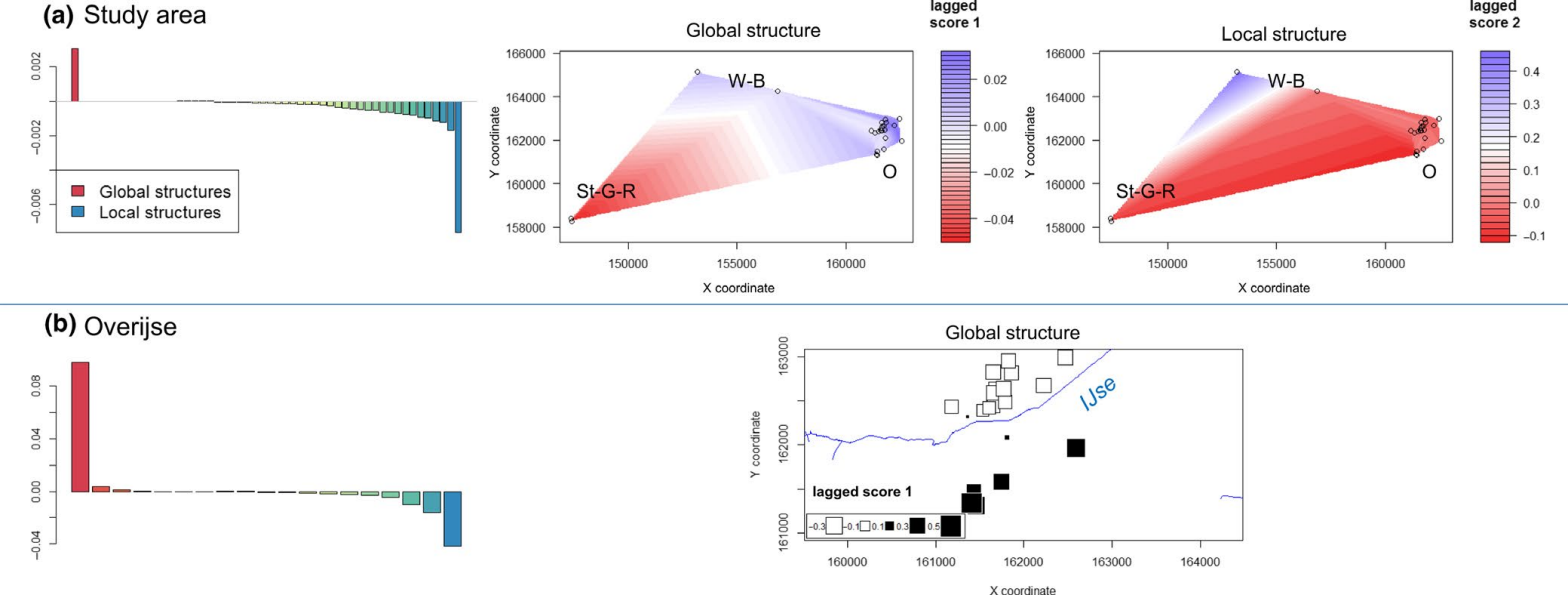
Spatial principal component analysis (sPCA) results for (a) all samples in the study area and (b) for Overijse separately, with barplots of the eigenvalues (left) and plots showing lagged scores of the largest global eigenvector (global structure) and of the largest local eigenvector (local structure) according to sampling location. The lagged scores were either (a) interpolated or (b) plotted as squares with size and color scaled relative to lagged scores. The eigenvalues in the barplot for all samples (a) are those using jittered coordinates (min. 1 m and max. 5 m). W‐B: Watermaal‐Bosvoorde; O: Overijse; St‐G‐R: Sint‐Genesius‐Rode

The PCA results showed Watermaal‐Bosvoorde to be distinct from the other two locations but with overlap, whereas Overijse and Sint‐Genesius‐Rode appeared to be very similar (Figure 5). The sample taken near Hoeilaart in 2015 was positioned intermediately between the three populations, although slightly closer to the center of the Watermaal‐Bosvoorde cluster (Figure 5). The sPCA resulted in only one component with positive eigenvalues. This component identified a global structure where the samples from Sint‐Genesius‐Rode were differentiated from the other samples (Figure 6a). This global structure was however not significant (*p* = .122). When we jittered the coordinates maximally 5 m and minimally 1 m, this global structure became more important with larger eigenvalues and became significant (*p* = .001; Figure 6a). One component with highly negative eigenvalues was also of importance, representing the local structure (*p* = .001, *p* = .039 after jittering) differentiating the Watermaal‐Bosvoorde population from the other two. The female stag beetle found near Hoeilaart was according to this analysis more related to the population in Overijse. In addition, a local structure in Overijse was present, which was more clear when we performed the sPCA analysis solely on samples from Overijse (Figure 6b). The global structure in Overijse due to the first principal component (*p* = .023) resulted in a division of samples in two groups, north and south of the valley of the river IJse.

**FIGURE 7:**
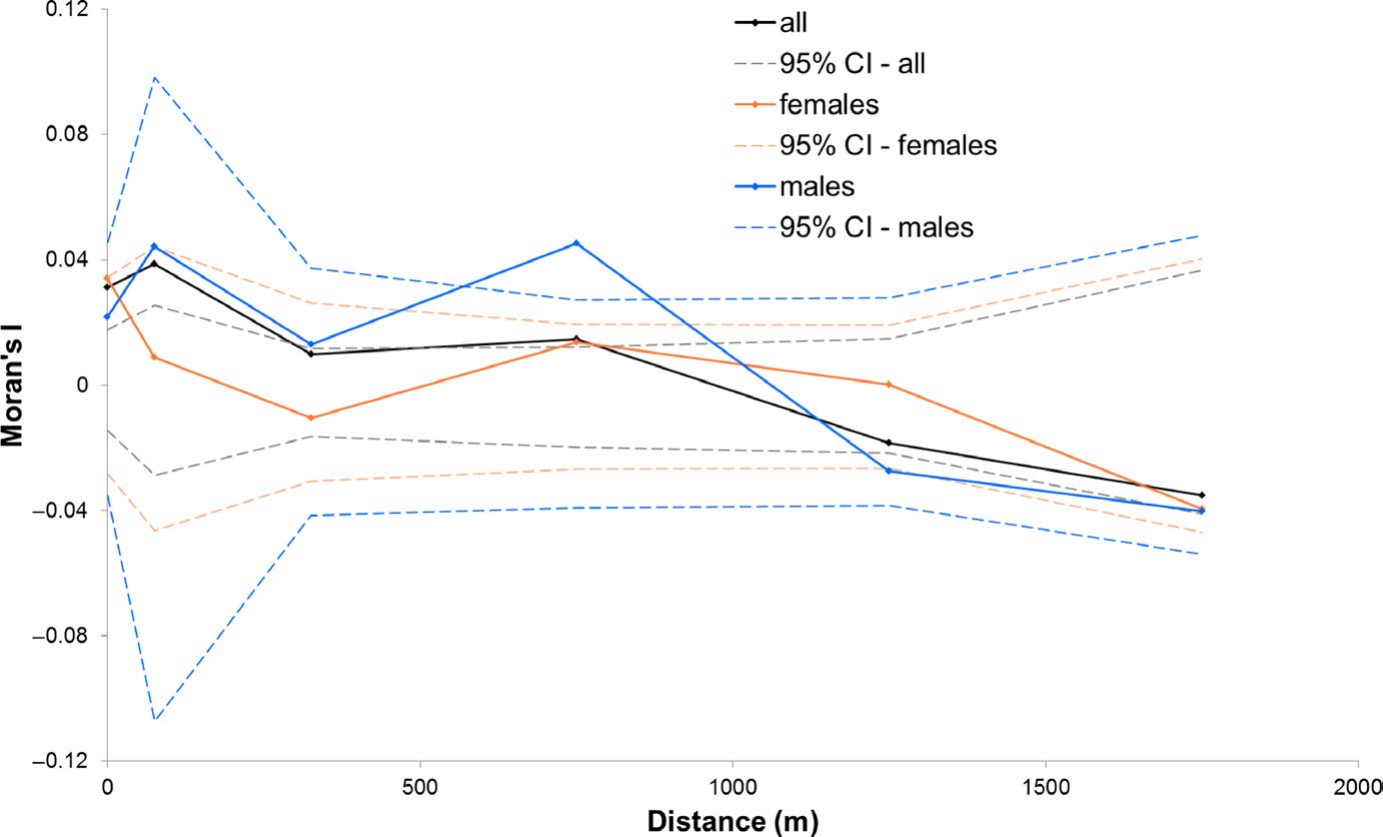
Spatial autocorrelation of *Moran's I* and geographic distance for stag beetles sampled in Overijse from 2007 to 2009 (black), for the female stag beetles (orange) and males (blue). 95% confidence intervals (dashed lines) are calculated by permuting individual locations among all individuals under the null hypothesis that genotypes of all adults are distributed randomly

Although highly significant (*p* = 0), the estimated coefficient of the geographic distances in the IBD model including all individuals was very low (β = 6.27E‐05, *SE* = 1.10E‐06, *t* = 57.06), as was the pseudo‐*R* ^2^ (0.075). The pseudo‐*R* ^2^ became even smaller (0.007) when only the samples of Overijse were included in the model (*β* = 1.36E‐04, *SE* = 1.52E‐05, *t* = 8.97, *p* = 0). Spatial autocorrelation was significant in the first distance class with a maximum distance of 150 m and within the class of 500 m to 1 km (Figure 7).

Under the *L*_home_ */L*_max_ criterion in GENECLASS2, three immigrants during the last generation were found in Overijse with two from Watermaal‐Bosvoorde (*p* = .004 and *p* = .002) and one from Sint‐Genesius‐Rode (*p* = .008), one in Sint‐Genesius‐Rode from Watermaal‐Bosvoorde (*p* = .002) and two in Watermaal‐Bosvoorde with one coming from each of the other populations (*p* = 0 and *p* = .007). Three of these immigrants were female, and the sex of three remaining individuals was unknown. If we assumed the *L_home_* criterion, only one immigrant in Overijse from Watermaal‐Bosvoorde (*p* = .008) was detected. According to the BAPS results for the same period (from 2008 to 2009), the same three immigrants coming from Watermaal‐Bosvoorde as identified under the *L*_home_ */L*_max_ criterion were assigned to the cluster of Watermaal‐Bosvoorde. Besides these three immigrants in Overijse and Sint‐Genesius‐Rode, three other individuals were sampled in Overijse but assigned to Sint‐Genesius‐Rode using BAPS. Another two individuals sampled in Sint‐Genesius‐Rode were assigned to Watermaal‐Bosvoorde. Of all of these migrant individuals one was male, two female and the remaining of unknown sex.

When we searched for full‐sibs among different sampling years, 11 full‐sib pairs were found when all samples were considered as offspring. Individuals of eight of the full‐sib pairs were sampled in the same year and at the exact same location (with 9 males, 5 females, and 5 unknown sex). Individuals of two full‐sib pairs were sampled with one year difference (2008 and 2009) at different locations in Overijse. For one of these full‐sib pairs (with one male and one of unknown sex), this resulted in a distance of 202 m between locations, for the other pair (with one male and one female) in a distance of ca. 1,236 m. Finally, there is one full‐sib pair with individuals sampled in 2002 and 2008 in Watermaal‐Bosvoorde, in the Jagersveld. When subsets by location and year were analyzed as offspring with individuals collected in earlier years as potential parents, the same full‐sib pairs were identified with a probability > .95 and none of the parental genotypes were assigned as mother or father of the investigated offspring.

### 3.3 Sex‐biased dispersal

Spatial autocorrelation analysis for samples collected during 2007–2009 in Overijse showed no significant relationship between *Moran's I* and geographic distance for the female stag beetles (Figure 7). For males, spatial autocorrelation was significant in the third distance class, from 500 to 1,000 m. The regression slope on the logarithm of spatial distance was only significant for the males (*b* = −0.005, *R^2^* = 0.0002, *P obs < exp* =.0098), not for the females (*b* = −0.037, *R^2^* = 0.013, *P obs < exp* = .294).

Table 3 shows the values for *mAIc*, *vAIc*, *F*_ST_ and *F*_IS_. The values for the different statistics point mostly toward the females as the dispersing sex, except *F*_IS_ which is mostly higher in the male group. Nevertheless, almost all *p*‐values for the randomization tests are higher than 0.05. Only the *F*_ST_ value for Watermaal‐Bosvoorde and Overijse in 2008 was significantly higher for the males (*p* = .0421) and *mAIc* was significantly lower for the females from Overijse in 2016 (*p* = .032) and for the females when analysing all samples together (*p* = .0404).

**TABLE 3:** Results of the randomization tests for sex‐biased dispersal

Sample	Sex	*N*	*mAIc*	*vAIc*	*F*_ST_	*F*_IS_
All populations, all years	Female	118	−0.4156	15.8556	0.0528	0.0590
	Male	135	0.3629	13.2150	0.0750	0.0829
	*p*		.0404	>.1	.0658	>.1
Overijse, Watermaal‐Bosvoorde (2008)	Female	51	−0.570	12.8071	0.0552	0.0508
	Male	38	0.7645	11.393	0.1004	0.0784
	*p*		.0406	>.1	.0339	>.1
Overijse, Sint‐Genesius‐Rode (2009)	Female	22	0.4029	14.0859	0.0608	0.0053
	Male	26	−0.4762	8.6858	0.0595	0.0056
	*p*		>.1	>.1	>.1	>.1
Overijse, all years	Female	63	−0.5063	18.6218		
	Male	59	0.5407	14.8244		
	*p*		.0817	>.1		
Overijse (2008)	Female	25	−0.7794	16.6567		
	Male	17	1.1461	14.8795		
	*p*		.0685	>.1		
Overijse (2009)	Female	23	0.4919	13.9287		
	Male	19	−0.5954	10.9867		
	*p*		>.1	>.1		
Overijse (2015)	Female	7	−0.4613	12.8800		
	Male	5	0.6458	6.7500		
	*p*		>.1	>.1		
Overijse (2016)	Female	5	−2.8378	21.9384		
	Male	9	1.5767	7.9188		
	*p*		.032	>.1		
Watermaal‐Bosvoorde, all years	Female	50	−0.1682	18.9093		
	Male	71	0.1184	15.3186		
	*p*		>.1	>.1		
Watermaal‐Bosvoorde (2002)	Female	9	−0.3373	18.8491		
	Male	17	0.1786	13.8075		
	*p*		>.1	>.1		
Watermaal‐Bosvoorde (2005)	Female	15	−0.5160	10.1350		
	Male	33	0.2345	12.7826		
	*p*		>.1	>.1		
Watermaal‐Bosvoorde (2008)	Female	26	−0.1994	8.6446		
	Male	21	0.2469	12.6415		
	*p*		>.1	>.1		

*N*, the number of samples per sex; *mAIc*, mean assignment index; *p,* the *p*‐values for the respective randomization tests (one‐sided); *vAIc*, variance of the assignment scores.

The relatedness among females (*r* = 0.062) was significantly lower than among males (*r* = 0.082) in Watermaal‐Bosvoorde when all sampling years were analyzed together (*p* < .02). The difference in relatedness in Overijse for males and females was slightly higher (*∆r* = 0.059), as mean relatedness was −0.001 among females and 0.058 among males, for all samples together (*p* < .02). In 2008, males were also more related to each other than females in Overijse (male *r* = 0.098, female *r* = −0.019, mean *∆r* = 0.117; *p* < .02). This was also the case for Overijse in 2009 (male *r* = 0.062, female *r* = 0.011, mean *∆r* = 0.051; .02 < *p* < .05). No significant difference among both sexes in relatedness was found for other combinations of sampling year and location. The analysis was not performed for Sint‐Genesius‐Rode because of its low sample size.

### 3.4 Effective population size and bottlenecks

The single sample estimates of *N_e_* are given in Table 4. Values for *N_e_* are low using the sibship method (10–36). The LDNE estimates vary more from very low (2 for Sint‐Genesius‐Rode in 2009) to relatively high (107 for Overijse in 2009). Temporal *N_e_* estimated with the Jorde and Ryman (2007) approach were low in Watermaal‐Bosvoorde despite the number of generations considered (9–33), while they were quite high in Overijse, although very variable (51–342) (Table 5). Using the maximum likelihood approach, MLNe, to estimate *N_e_* over different time periods, the values for Overijse varied from 89 to 527, but always with the maximum possible *N_e_* as upper limit of the confidence intervals (Table 6). Gilbert and Whitlock (2015) found that while MLNe provides accurate estimates of *N_e_*, the coverage probability of the confidence intervals were inaccurate. When Overijse was assumed to be isolated, no estimate for *N_e_* below the assumed maximum *N_e_* was obtained. Using Watermaal‐Bosvoorde as source population usually resulted in much lower estimates than when Sint‐Genesius‐Rode acted as source population. Estimates for Watermaal‐Bosvoorde were stable among migration scenarios and were comparable to those obtained by the other methods, although *N_e_* estimated with temporal methods concerns a different time period than when using single sample methods.

**TABLE 4:** Single sample estimates of *N_e_* using the linkage disequilibrium and sibship approach

Population	Year	LD		Sibship	
		*N_e_*	CI	*N_e_*	CI
Watermaal‐Bosvoorde	2002	–	–	26	15–49
Watermaal‐Bosvoorde	2005	29.1	16.1–63.6	29	18–51
Watermaal‐Bosvoorde	2008	–	–	30	18–50
Sint‐Genesius‐Rode	2009	1.9	1.2–4.7	12	5–61
Overijse	2008	67.5	35.6–217.2	28	17–48
Overijse	2009	106.8	47.7–2,156.9	36	23–60
Overijse	2015	–	–	10	5–28
Overijse	2016	23.9	10.6–164	21	10–61

Abbreviations: CI, 95% confidence interval of *N_e_*; LD, linkage disequilibrium approach.

**TABLE 5:** Temporal estimates of *N_e_* using the Jorde and Ryman (2007) approach

Population	Sampling years	*N_e_*	CI
Watermaal‐Bosvoorde	2002–2005	8.8	5–13.6
Watermaal‐Bosvoorde	2005–2008	17.6	9.8–27.8
Watermaal‐Bosvoorde	2002–2008	32.7	18.3–51.1
Overijse	2008–2015	135.8	81.3–204.2
Overijse	2008–2016	342.4	206.3–512.4
Overijse	2009–2015	50.5	30.4–75.6
Overijse	2009–2016	–	–

Abbreviation: CI, parametric 95% confidence interval of *N_e_*.

**TABLE 6:** Results for *N_e_* using MLNE with and without migration. The number of generations was defined as two

Population	Sampling years	m from St‐G‐R	m from W‐B	m from O	*N_e_*	CI
Overijse	2008‐2015				*N_e_* max	73.11 ‐> *N_e_* max
Overijse	2008‐2015	x			*N_e_* max	57.47 ‐> *N_e_* max
Overijse	2008‐2015		x		437.37	43.65 ‐> *N_e_* max
Overijse	2009‐2015				*N_e_* max	61.40 ‐> *N_e_* max
Overijse	2009‐2015	x			216.20	31.634 ‐> *N_e_* max
Overijse	2009‐2015		x		116.17	31.81 ‐> *N_e_* max
Overijse	2008‐2016				276.25	40.53 ‐> *N_e_* max
Overijse	2008‐2016	x			136.58	29.59 ‐> *N_e_* max
Overijse	2008‐2016		x		89.45	27.59 ‐> *N_e_* max
Overijse	2009‐2016				*N_e_* max	51.00 ‐> *N_e_* max
Overijse	2009‐2016	x			526.97	37.88 ‐> *N_e_* max
Overijse	2009‐2016		x		131.64	32.66 ‐> *N_e_* max
Watermaal‐Bosvoorde	2002‐2005‐2008				27.29	16.52 – 56.95
Watermaal‐Bosvoorde	2002‐2005‐2008	x			28.93	17.81 – 54.74
Watermaal‐Bosvoorde	2002‐2005‐2008			x	29.92	18.52 – 54.59

Abbreviations: CI, confidence interval for *N_e_*; m, migration; *N_e_* max, maximum effective population size set as 10,000; O, Overijse; St‐G‐R, Sint‐Genesius‐Rode; W‐B, Watermaal‐Bosvoorde.

A significant signal for a recent genetic bottleneck was found under the IAM model for all populations and years, except for Overijse in 2015 (Table 7). The Wilcoxon signed rank test performed using the TPM model was only significant for Watermaal‐Bosvoorde. The allele frequency distribution showed a mode shift distortion in 2016 for the samples of Overijse which suggests the occurrence of a recent bottleneck.

**TABLE 7:** Results of Bottleneck analysis. The *p*‐values of the Wilcoxon signed rank test are given for two mutation models. The shape of the allele frequency distribution is also given

Population	Year	*p*‐value Wilcoxon signed rank test		Mode allele frequency distribution
		IAM	TPM	
Watermaal‐Bosvoorde	2002	.00011	.02884	L‐shaped
Watermaal‐Bosvoorde	2005	.00004	.00775	L‐shaped
Watermaal‐Bosvoorde	2008	.00002	.00258	L‐shaped
Overijse	2008	.00084	.39098	L‐shaped
Overijse	2009	.00019	.33427	L‐shaped
Overijse	2015	.20187	.88440	L‐shaped
Overijse	2016	.00105	.21660	shifted mode

Abbreviations: IAM, the infinite alleles model; TPM, two phase model.

## 4 DISCUSSION

There have been numerous studies of the effects of habitat fragmentation on genetic diversity in animals; however, this has been rarely studied among insects. In general, insects represent a group that has received scant study of fine scale spatiotemporal population processes (but see Bretman et al., 2011; López‐Uribe et al., 2015; Melosik et al., 2020; Oleksa et al., 2015; Schauer et al., 2018; Zytynska et al., 2018). This study assessed fine scale spatiotemporal genetic variation within and among habitat patches that have been recently fragmented by urbanization using a suite of *F*_ST_, individual assignment, and kinship analyses. The results revealed numerically small to moderate but statistically significant genetic differentiation among patches. A key feature was that this differentiation was evident over multiple temporal samples and therefore indicates “biologically meaningful” restricted gene flow (Waples, 1998). In addition to restricted gene flow, assignment and kinship analyses confirm that contemporary dispersal between patches is highly restricted. Firstly, clustering analyses delineated groups that aligned with patch membership. Secondly, assignment analyses revealed low rates of migration. Collectively these results indicate that the recently fragmented areas now represent demographically independent populations from one another, at least over decadal time scales of relevance to conservation. Bottleneck and *N_e_* estimators suggest recent genetic declines and local populations to comprise small numbers of individuals that are successfully breeding. There were also indications of further spatial structuring within sites and social cohesion.

### 4.1 Population genetic structure

Population genetic studies of saproxylic insects have typically reported weak genetic structuring over larger geographical ranges than studied here (Komonen & Muller, 2018). Similarly, Cox et al. (2019) reported low levels of genetic structure across European stag beetle. This weak genetic structure has often been interpreted as indicating that the species have better than thought dispersal abilities and/or that there exist enough connectivity corridors between populations. However, an important consideration is that many populations may not be at migration‐drift equilibrium, in which case genetic differentiation as estimated by *F*_ST_, *G*_ST_ or *D*_est_ may reflect historical gene flow rather than contemporary dispersal rates (Jost, 2008; Ryman & Leimar, 2009). The mtDNA analysis, specifically mismatch and neutrality test results from Cox et al. (2019), indicates that most stag beetle populations have not yet attained equilibrium following postglacial colonization. The attainment of equilibrium is expected to be even slower at diploid nuclear loci compared to mtDNA. Assignment tests have proven extremely useful in elucidating patterns of restricted dispersal even in such nonequilibrium systems (e.g., Castric & Bernatchez, 2004) and here both BAPS and classical assignment tests reported low levels of dispersal between these regions. Accordingly we posit that the low to moderate *F*_ST_ reported here, rather than reflecting gene flow between patches, reflects nascent incipient differentiation among recently isolated demes. As the fragmentation of populations in the area occurred mainly between 1930 and 1970, the short time frame may have limited the accumulation of genetic differentiation among the relict populations. The lower differentiation estimates among Overijse and Sint‐Genesius‐Rode could suggest that gene flow occurred more frequently south of the Sonian Forest, since stag beetle prefers half open habitats as opposed to closed canopy (Thomaes et al., 2010).

There was no clear temporal structuring for either of two patches Overijse and Watermaal‐Bosvoorde. Genetic differentiation between years was largely nonsignificant. Likewise, Snegin (2014) found genetic similarity among groups of individuals sampled in consecutive years to be very high. In Overijse, only one comparison of relatedness within a year and among years was significantly different from zero. For Watermaal‐Bosvoorde, there were more comparisons significant, but always one of two possible comparisons for each pair of years (i.e., the comparison of relatedness within year 1 or year 2 with relatedness between year 1 and year 2). Full‐sibs sampled a year apart were, furthermore, found in Overijse. Another full‐sib pair was detected in Watermaal‐Bosvoorde sampled six years apart. Analysis results on subsets with offspring and potential parental genotypes suggested this full‐sib relationship was not mistaken for a parent‐offspring relationship. Although an increase of the larval stage by six years is highly unlikely, this finding supports a close relationship among sampling years. Collectively this indicates that the two patches for which sampling was sufficient to test temporal patterns, are largely self‐sustaining and not prone to extinction‐recolonization processes.

### 4.2 Fine scale structure

Understanding the role of geographical distance is important for defining management units. When we explored the local spatial structure in Overijse, no clear IBD pattern was present, while sPCA results showed a subtle division of northern and southern samples (all years together). This could have resulted from local, non‐clinical landscape features, as the alluvial valley of the river IJse is unsuitable for oviposition (Thomaes et al., 2008). Spatial autocorrelation analyses showed only significantly more related individuals within the sampling location (distance class 0–150 m) and again in the distance class of 500 m to 1 km. As eight of the eleven full‐sib pairs we identified were collected in the same year and location, this supported the social cohesion present within the smallest distance class. The 500 m to 1 km distance class coincides with the majority of maximum dispersal distances recorded for the species, but usually with higher distances for males than females (Rink & Sinsch, 2007; Thomaes, 2009). For the full siblings collected one year apart on different locations in Overijse, this resulted in distances between siblings of c. 202 m and 1,236 m. This could mean that eggs were laid at different locations by the same mother and/or that one or both full siblings dispersed in their adult stage. However, as the individuals were found as road casualties and as prey rests from birds, the beetles might have not covered these distances autonomously. They could have been carried away by traffic or birds some distance before they were collected. Corvids, such as carrion crows (*Corvus corone*) and magpies (*Pica pica*), tend to kill them close to their site of emergence (Fremlin et al., 2012), preferably on open ground, such as on roads where potential predators are more visible and for mechanical reasons (Campanaro et al., 2011). Further research is needed with more detailed information to capture the cause of the local spatial pattern. However, it appears to be driven by local social behavior and landscape effects.

### 4.3 Sex‐biased dispersal

According to previous studies, female stag beetles exhibited smaller dispersal distances than males (Rink & Sinsch, 2007; Tini et al., 2018). Rink and Sinsch (2011) reported a mean dispersal distance of 203 m for females and of 802 m for males based on monitoring results collected over three years. We therefore expected the local structure to be mainly attributed to the limited dispersal of the females. Still, females were not more related to each other than was expected at random. On the other hand, the male stag beetles showed some spatial autocorrelation. On a population level, assignment index metrics and relatedness within populations also indicated the female stag beetle to be the dispersive sex, or at least provided no evidence for significant lower female dispersal distances. When Overijse and Watermaal‐Bosvoorde were analyzed together, dispersal appeared again to be mainly female‐biased, with *mAIc* and/or *F*_ST_ being significantly lower for females than males. Though the sex of the immigrants detected using GENECLASS2 was partly unknown, three of them were female, as was the individual found near Hoeilaart. The origin of this beetle is unclear and could either be Watermaal‐Bosvoorde or Overijse. Still, this suggests that this female has crossed at least 3.8 km. In order to colonize new oviposition sites females might be inclined to disperse over longer distances, depending on the spatial distribution of suitable dead wood. Furthermore, female stag beetles have a greater tendency to fly when they want to reach more open canopy (Thomaes et al., 2018). While female stag beetles may stay close to their site of emergence when suitable oviposition sites are abundant and nearby (Tini et al., 2018), they can traverse larger distances when such sites are rather scarce or already occupied by other larvae (Harvey, 2007). Long‐distance dispersal across generations was also suggested by Cox et al. (2019) on the basis of the maternally inherited mtDNA. A possible explanation for the discrepancy between the genetic and radio‐telemetric outcomes is that the telemetric studies did not compensate for the longer female activity period (Harvey et al., 2011)(adult activity period is 2–3 weeks for males and up to 2 months for females in the Belgian populations (Thomaes et al., 2010)). In telemetric studies the initial period of adult activity after emergence is followed, as battery lifetime is limited to 10–15 days.

### 4.4 Genetic bottleneck

In this study we employed heterozygosity excess and mode shift tests to identify population declines occurring over recent time scales. Specifically, for heterozygosity excess it is estimated that bottleneck footprints will relate to events occurring with <4*N_e_* generations, while for mode shift this time frame is even shorter (Cornuet & Luikart, 1996). Heterozygosity excess tests revealed significant bottleneck signatures for both IAM and TPM for all Watermaal‐Bosvoorde tests. Significant heterozygosity results were also reported under the IAM in a number of cases for Overijse (2008, 2009, 2016), with the 2016 sample also reporting a significant mode shift. In general the biggest criticisms of the bottleneck detection tests employed here, and other such tests, have been their high Type II error with genetic studies often failing to identify bottlenecks despite compelling demographic data (Busch et al., 2007; Le Page et al., 2000; Mardulyn et al., 2008; Peery et al., 2012; Queney et al., 2000; Steinfartz et al., 2007). Furthermore, it is important to note that the detection of a recent bottleneck may be compromised in situations where ancestral diversity was already low, as suggested for European stag beetle (Cox et al., 2019). Therefore, significant results reported here strongly support the populations have both experienced or are experiencing recent genetic bottlenecks.

Small population size is an important factor in loss of genetic diversity, exacerbated by genetic bottlenecks, which may increase the risk of inbreeding (Allendorf et al., 2013). Here we generated estimates of contemporary effective (or breeding) population sizes using two temporal methods and two single samples estimates (LD and sibship). Although stag beetle was believed to be a species with discrete generations, our results seemed to suggest otherwise. Stag beetles might be considered more as a semelparous species with variable age at maturity, described as the Salmon‐model by Waples (2005). In this case, single sample estimates are of *N_b_* (effective number of breeders) and are affected by *N_b_* in prior years when sampling adults. Estimates of *N_b_* derived by the temporal methods, on the other hand, apply to a number of years prior to both sampling years (Waples, 2005).

Estimates of *N_e_* or rather of *N_b_* were generally small, particularly for Sint‐Genesius‐Rode and Watermaal‐Bosvoorde. In Watermaal‐Bosvoorde, *N_b_* estimates were similar across methodologies. Monitoring of stag beetle in Watermaal‐Bosvoorde started in 1962 (R. Cammaerts, unpublished data) and showed a continuous population present. Initially Watermaal‐Bosvoorde held only one stag beetle population, in a forested slope a few 100 meters north of Jagersveld. It was assumed Jagersveld and other nearby sites were colonized by stag beetles from that particular slope. Jagersveld became eligible, man‐made habitat for stag beetle when oak logs for fencing were incorporated in a school garden. The clear signs of a reduction in population size could therefore be attributed, at least partly, to a founder event instead of a bottleneck. The lowest *N_b_* estimate was found for Sint‐Genesius‐Rode. Despite the low sample size, which obliged us to use only the single sample estimators, the estimate of *N_b_* is in agreement with the lower gene diversity found in the population. Overijse holds the highest gene diversity and allelic richness of the three populations, though slightly lower levels than those found in surrounding areas of West Europe (Cox et al., 2019).

### 4.5 Implications for conservation

The data indicate that the three sampled areas are reciprocally isolated in terms of dispersal and gene flow. Furthermore, populations may comprise limited numbers of successfully breeding individuals and exhibit signatures of recent demographic declines. As such, these populations must be considered vulnerable and managed separately. The reported isolation means that rescue events may be limited and/or unpredictable. Small, recently isolated populations are at risk of reduced viability owing to demographic and genetic (inbreeding) effects which can lead to such populations entering an “extinction vortex” (Gilpin & Soulé, 1986). In this case, if one population crashes the available habitat patch may not be readily recolonized, which in turn could have a domino effect on the persistence of other populations within a metapopulation system. Conservation efforts should focus on increasing habitats (dead wood with the necessary fungi) within and between populations. The lack of such habitats within the suburban landscape here may be a driver of the slight female bias in dispersal as they search for oviposition sites.

## CONFLICT OF INTEREST

None declared.

## AUTHOR CONTRIBUTION

**Karen Cox:** Conceptualization (equal); Formal analysis (lead); Investigation (lead); Validation (lead); Writing‐original draft (lead); Writing‐review & editing (lead). **Niall Mckeown:** Writing‐review & editing (equal). **An Vanden Broeck:** Writing‐review & editing (equal). **An Van Breusegem:** Investigation (supporting); Resources (equal); Writing‐review & editing (supporting). **Roger Cammaerts:** Resources (equal); Writing‐review & editing (equal). **Arno Thomaes:** Conceptualization (equal); Resources (equal); Writing‐review & editing (equal).

## Data Availability

Microsatellite genotypes were deposited in Dryad: https://doi.org/10.5061/dryad.573n5tb5h.
